# Factors affecting the learning curve in robotic colorectal surgery

**DOI:** 10.1007/s11701-022-01373-1

**Published:** 2022-02-01

**Authors:** Shing Wai Wong, Philip Crowe

**Affiliations:** 1 Department of General Surgery, Prince of Wales Hospital, Sydney, NSW Australia; 2 Prince of Wales Clinical School, The University of New South Wales, Sydney, NSW Australia

**Keywords:** Learning curve, Robotic colorectal surgery, Operative time, Multidimensional analysis

## Abstract

Learning related to robotic colorectal surgery can be measured by surgical process (such as time or adequacy of resection) or patient outcome (such as morbidity or quality of life). Time based metrics are the most commonly used variables to assess the learning curve because of ease of analysis. With analysis of the learning curve, there are factors which need to be considered because they may have a direct impact on operative times or may be surrogate markers of clinical effectiveness (unrelated to times). Variables which may impact on operation time include surgery case mix, hybrid technique, laparoscopic and open colorectal surgery experience, robotic surgical simulator training, technology, operating room team, and case complexity. Multidimensional analysis can address multiple indicators of surgical performance and include variables such as conversion rate, complications, oncological outcome and functional outcome. Analysis of patient outcome and/or global assessment of robotic skills may be the most reliable methods to assess the learning curve.

## Introduction

The exponential increase in adoption of robotics for colorectal surgery has occurred concurrently with improvements in technology. This may be related to improved ergonomics for the operating surgeon with benefits in the areas of visualisation, posture and manipulation [1]. The learning curve describes the rate of progress in acquiring new skills and has implications for training, self-assessment, credentialing, clinical outcomes and cost–benefit decision regarding adoption of new procedures and devices [2]. Learning related to a new surgical technique can be measured by surgical process or patient outcome [3]. Surgical process measures are generally easier to analyse and include factors such as operation time and adequacy of resection for cancer surgery. Patient outcomes include operative morbidity, patient satisfaction and quality of life.

Other variables should also be taken into account when the learning curve is assessed. Some of these factors may have a direct impact on operative times and other factors may be surrogate markers of clinical effectiveness (unrelated to times). Variables which may impact on operation times include operation case mix (selected or sequential cases), hybrid technique, prior laparoscopic and open surgery colorectal surgery (LCS and OCS) experience, robotic surgical simulator training, advances in technology, dedicated operating room team and case complexity. Harryson et al., in their systemic review of learning curves for minimally invasive abdominal surgery, concluded that an ideal analysis should account for the degree of complexity of individual cases and the inherent differences between surgeons [4].

Multidimensional analysis can address multiple indicators of surgical performance and include variables such as conversion rate, complications, oncological outcome and functional outcome. Multidimensional analysis of all significant variables would be more representative of a new technique’s learning curve because a reduction in operation time can occur at the expense of optimal patient outcome [5]. Similarly, global assessment of robotic skills may be a better measure of surgeon expertise than time improvements. The aim of the review is to examine the literature concerning factors which may impact the learning curve in robotic colorectal surgery, either by directly influencing operative times (Table 1) or by analysis of non-time based outcomes (Table 2).

**Table 1:** Factors affecting operation time

Factors	Assessment
Case number	Chronological order Moving average method Cumulative sum analysis (CUSUM)
Surgery case mix	All consecutive cases One type of surgery only (e.g. rectal)
Hybrid surgery	Laparoscopic mobilisation of part of colon Total robotic approach
Experience	Open colorectal surgery Laparoscopic colorectal surgery
Surgical simulator training	Proficiency-based learning
Advanced technology	daVinci Si or Xi robot
Standardised surgical protocol	Dedicated team
Case complexity	Body mass index Colon or rectal resection Cancer tumour stage Female or male pelvic case Extent of inflammation

**Table 2:** Learning curve assessment unrelated to time analysis

Indicators of surgical performance	Variables
Surgical outcome	Conversion rate Perioperative morbidity
Oncological outcome	Adequacy of resection Lymph node harvest Local recurrence
Functional outcome	Patient satisfaction Quality of life
Global assessment of robotic skills	Depth perception Bimanual dexterity Efficiency Force sensitivity Autonomy Robotic control

## Case number

Studies have reported wide variation in the length of learning curves. In LCS, studies indicated that a learning curve of 40–70 cases is required for proficiency [6]. Most studies report a plateauing of the learning curve for robotic colorectal surgery after 15–20 cases [7, 8]. More complicated rectal dissections may have a bimodal plateau after 33–45 cases and again at 72–93 cases [9–11]. A systematic review of 28 LCS and 6 RCS studies indicated the number of operations to achieve proficiency to range from 5 to 310 cases for LCS and 15–30 cases for RCS [12]. The authors noted that the definition of proficiency on the basis of single parameters was useful but simplistic. Robotic surgery may attenuate the steeper learning curve of laparoscopic surgery by improving dexterity and efficiency. The learning curve for a novice rectal surgeon was noted to be 25 cases and was attributed to flattening of the steep learning curve by the surgical robotic system, perhaps not taking into account simulation training [13].

The learning curve has historically been described as having four phases: rapid ascent, slower ascent, plateau or asymptote, and descent [3]. The rapid ascent indicates how quickly individuals learn and master stages of a complex procedure before a competent point is reached. Additional experience improves outcomes by small amounts until a plateau is reached. Sometimes a temporary performance deterioration is seen during this phase, which may be related to a more difficult case mix or over-confidence. The final phase of decent occurs much later with advancing age and deterioration in manual dexterity, eyesight and cognition.

Time based metrics have been the most commonly used variables to assess the learning curve [2]. Time changes during the learning curve of RCS has been evaluated by raw times plotted in chronological order, the moving average method and cumulative sum (CUSUM) analysis. The moving average method is created by an average of subsets (e.g. a moving average order of 20), which are modified by adding new data to the subsets and then by shifting forward all data set. This method can present the overall trend of data to detect cumulative changes from average values [8, 11]. The results from a study including this method and CUSUM analysis revealed similar patterns with low peak and high peak points at the same chronological case numbers [11].

CUSUM analysis can be used for monitoring performance and detecting areas of improvement. The CUSUM is the running total of differences between the individual data points and the mean of all data points. This allows visualisation of data for trends not discernible with other approaches. The advantages of CUSUM analysis are independence from sample size, effectiveness in detecting small shifts in the system and ability to allow continuous analysis in time and rapid evaluation of data [14].

Three distinct performance phases with CUSUM analysis of surgeon console time (SCT) have been described during RCS. The initial phase typically has a positive slope which is associated with the longest console time (attributed to learning), followed by a plateau (attributed to competency), and finally a negative slope (attributed to mastery). However, the studies were not always consistent in their presentation of the three phases, with some studies displaying different orientation of the learning curve slopes (i.e. descent followed by plateau and then ascent).

The most typical positive slope/ plateau/ negative slope pattern with CUSUM graphical analysis was demonstrated in several RCS studies [14, 15]. The CUSUM graph of two other studies had an initial negative slope, followed by plateau and then positive slope [7, 16]. One study attributed the positive slope to more complicated cases during phase 3 [7]. In the other study, the decrease operative times over the three phases did not align with this slope pattern [16]. The surgeons in these studies may have not achieved mastery (and possibly not competency) because the CUSUM curves at the end of the cases were still ascending. The CUSUM robotic operation time curve of experienced laparoscopic colorectal surgeons in two studies indicated a gradual decline of the console time CUSUM curve for the first 44/45 cases, perhaps demonstrating minimal learning process. [17, 18].

## Surgery case mix

Two studies which included a heterogeneous group of consecutive colorectal procedures indicated that 83 and 88 cases were required to attain mastery [15, 18]. The longer time to attain mastery may be related to the fact that all consecutive patients who underwent RCS were included in the studies. Learning may be facilitated when performing a variety of bowel resections. Some procedures are inherently more complex and challenging than others. However, each case type has a unique set of steps, instrument use and skill sets [15]. The case numbers required to complete the competency phase differ according to the consecutiveness of case mix, which may lead to selection bias. Most studies were single surgical procedure studies and do not account for learning during other procedures in between these nominated cases. The number of cases to reach competency for one type of surgery only (e.g. rectal) would be less because not all completed RCS cases have been included in the analysis.

Subgroup analysis has demonstrated different learning curves when certain case types are selected from consecutive RCS cases. One RCS study with a study population of 62 patients, compared the results before and after the 15th case [8]. There was non-significant increase in total operation time (TOT) from 261 to 312 min for the 11 patients who underwent robotic right hemicolectomy, significant reduction in TOT from 361 to 258 min (*p* = 0.03) for the 14 patients who underwent robotic rectopexy, and significant reduction from 518 to 395 min (*p* = 0.02) for the 24 patients who underwent robotic proctectomy for rectal cancer. The improvement in time associated with experience in the group of patients undergoing rectal surgery may have been related to skills learnt during robotic colon surgery cases. Another study examined the consecutive SCT CUSUM graphs of all colorectal cases and of selected case types (right hemicolectomy and rectal surgery) [18]. Unlike the graph displaying CUSUM of surgeon console time for all cases, the graphs for the selected surgery cases did not reveal any evidence of consistent upward or downward slope to indicate a learning process.

## Hybrid surgery

Hybrid procedures involve laparoscopic mobilisation of part of the colon (typically splenic flexure and descending colon) and completion of the surgery with robotic assistance (typically sigmoid colon and rectal dissection). This was more common with use of the older da Vinci S/Si systems which did not allow easy multi-quadrant surgery. Park et al. argued that there should be no difference between the learning curve of the totally robotic approach and the hybrid technique of robotic rectal cancer surgery because manipulating robotic instruments in a narrow pelvic space was the most challenging part to learn [10]. However, SCT would be shorter and achieving competency should involve less cases as less skill sets need to be mastered when performing only the pelvic part of the surgery robotically.

Comparing the mean SCT to the mean TOT can indicate how much of the operation was performed laparoscopically or via the open approach. Typically, the ratio is less than half with hybrid procedures and around two-thirds for total robotic surgery. Two studies using the total robotic approach on the da Vinci Xi system reported ratios of 0.64 (180/280) and 0.71 (214/302) [14, 18]. Sng et al. performed total robotic dissection using the da VInci S robotic system, which required repositioning of the robotic arms between the two phases of surgery [9]. Their ratio was 0.5 (140/279). Studies using a hybrid approach revealed ratios of 0.47 (115/246), 0.39(82/212), and 0.32(64/200) [7, 11, 19].

One study included 39 patients who had hybrid or total robotic approaches for the management of rectal cancer [13]. More total robotic cases were performed with later phases but this was surprisingly not reflected by an increase in the SCT/TOT ratio. Even in third phase when 7 of 14 patients had “total” robotic surgery, the mean ratio was 0.34, which would suggest a large proportion of the surgery was performed laparoscopically or open.

## Experience

The surgeon’s previous experience may be a significant factor in the variability of the operating time learning curves. Some studies have shown that surgeons with greater experience require fewer robotic procedures to overcome their learning curves [2]. Odermatt et al. found in their study of two surgeons that prior experience in laparoscopic rectal surgery had an impact on the learning curve for robotic rectal resections [17]. For the surgeon with extensive laparoscopic experience (1500 LCS procedures prior to starting robotic surgery), the CUSUM curves revealed minimal to no learning process for operation time and quality indicators such as lymph node harvest, length of stay and major complications. For the surgeon with less LCS experience (400 prior to starting robotic surgery), the CUSUM curves showed a clear learning process for operation time, length of stay (LOS) and major complications.

The main benefit of experience may be reflected in the low complication rate during the learning curve, rather than a reduction in TOT. Two studies reporting on RCS learning curve of consecutive unselected colorectal procedures reported different complication rates [15, 18]. The gastrointestinal complication rate (which included small bowel obstruction, ileus, anastomotic leak and abdominal/pelvic abscess) was 0.37 and 0.06 per patient (sixfold difference) in the two studies, possibly related to the difference in operative experience of the surgeons.

Inexperience in OCS and LCS may impact the learning curve. Inexperience in either surgery may be associated with longer operation times, higher open conversion rates and more complications [13, 21]. However, the robotic surgical system may allow smoother transition from open to minimally invasive surgery. An experienced surgeon may be able to transfer open surgical skills to a minimally invasive setting quicker with the help of the robotic system because of the already acquired proficiency in tissue handling and anatomic knowledge, as well as avoiding the visual/proprioception disconnect from the fulcrum effect with laparoscopic surgery. Several studies reported a reduction in TOT during the first 20 RCS cases by experienced OCS surgeons with limited LCS experience, with a rapid transfer of surgical skills from open to the robotic approach [19–21].

The learning curve may be shorter in surgeons who are already proficient in OCS and LCS. Different skill sets are learnt from the two different approaches which can be transferable to a robotic setting. Familiarity with the instruments and foot pedals used in laparoscopic surgery and with the anatomy viewed through a video monitor can help when first performing robotic surgery [19]. Regained depth perception but reduced haptic feedback with the robotic system would help with the RCS learning curve when transitioning from LCS.

## Surgical simulator training

Robotic simulation training allows surgeons to develop and improve skills that are directly transferrable to the operating room, and provide a record to track their progress [22]. Volume-based learning can be replaced with proficiency-based learning, as metrics are used to measure progress rather than number of procedures or years in training. Simulation can provide advanced procedural-based training, and can function as a warm-up exercise prior to actual surgery [23]. Increased robotic simulation training should have an impact on shortening the learning curve but the efficacy of virtual simulators in the acquisition of skills to the standard required for safe clinical RCS has not been studied [24]. Most RCS learning curve studies did not report surgeon simulation training experience [2].

## Advanced technology

The ability to complete all cases with a total robotic approach without conversion may be related to the improved technology of the fourth-generation daVInci Xi. Multivariate analysis by Ozben et al. demonstrated that the Xi robot was an independent factor associated with a reduced console time [25]. Other studies reported shorter operating time using the Xi (compared with Si) for robotic colorectal surgery and TME as well as better ability to perform a fully robotic approach [26–28]. The advantages of the Xi include improved flexibility and manoeuvrability with overhead rotating architecture, slimmer boom-mounted arms, extended instrument reach, guided targeting and integrated auxiliary technology [29]. Despite having a longer active chain and more robotic joints, the accuracy of the Xi was similar to the Si system [30]. The main problem of studies comparing outcomes of the Si and Xi platform relate to chronology bias and “proficiency-gain effect”. [27].

## Standardised surgical protocol

A standardized surgical protocol for all RCS was implemented in one study which included a dedicated team of operating room staff, standard instrument use, routine sequential operative steps and participation of two surgeons when warranted [31]. This was associated with a significantly reduced TOT (431 vs 279 min, *p* < 0.01), open conversion and LOS. Another study reported shorter learning curves in surgeons who were in a systematic institutional program [16]. The authors attributed this improved institutional efficiency to standardisation of patient and robot positioning, port placement, appropriate use of instruments, conflict resolution and dedicated assistant.

## Case complexity

Differences in case complexity may have the greatest impact on SCT learning curves observed. Following initial improvement and subsequent stabilisation of performance, a decline is commonly observed. This may be related to the willingness of surgeons to take on more challenging and complex cases once they have mastered the simpler surgical cases. Studies of simulated robotic tasks have observed variable learning curves for training tasks of different complexity [2].

Bokhari et al. found increase console time during the “mastery” phase compared with the initial phases, which they attributed to a greater number of difficult cases during that period [7]. The “mastery” phase may not have been accurate because the CUSUM curve was still ascending in their third phase. There was a greater proportion of more technically challenging case, morbidly obese patients, male patients and low pelvic malignancies during the mastery phase. Sng et al. found not only a significant decrease in console time with experiences but also a significant increase in length of stay [10]. The authors attributed this finding to the increasing complexity of cases the surgeon performed during the later phases. Comparing phase I patients (first 35) with the later phase patients (next 162), there was a lower percentage of low rectal cancers (46% vs 64%), neoadjuvant chemoradiotherapy (3% vs 33%) and splenic flexure mobilisation (9% vs 57%).

Limiting the complexity of robotic colorectal resection cases in the initial training phase may improve patient outcome. One study reported no increase in morbidities over the learning phases with gradual performance of more complex cases such as low cancers and preoperative chemoradiation [19]. Miskovic et al. graded complexity of LCS cases on factors such as body mass index, colon or rectal resection, tumour stage of cancer, female or male pelvic case and extent of inflammation [32]. These factors are probably similar for RCS cases. Using the complexity score of Miskovic et al. Shaw et al. found an improvement in mean operating time of 53 min with robot-assisted colorectal surgery after 15 cases, despite an increase in complexity of cases (10% vs 34% level IV complex cases, *p* = 0.03) [8]. Using the same score, Muller et al. reported a major complication rate in patients with high complexity of 34.5% (10/29 patients) in the first time period and 10.5% (2/10 patients) in the second time period [33]. The authors recommended limiting the complexity of robotic colorectal resection cases in the initial training phase.

In one study, the “learning” phase (with increase SCT) occurred in phase 2 and may have been related to performance of more complicated multi-quadrant total colectomy cases [18]. In another study, seven of eight exceptionally long TOT (more than one standard deviation above mean TOT) occurred in the middle phase (17 of 39 patients) of the learning curve [13]. This was related to difficult cases and resulted in a significant increase in TOT compared with the other two phases.

## Multidimensional analysis

Defining the learning curve on the basis of single parameters is useful but simplistic, with multidimensional assessment (MDA) more reliable [12]. MDA of the learning curve has been used to evaluate additional variables to operative time such as conversion, perioperative morbidity, circumferential margin, harvested lymph nodes and local recurrence [11, 19, 32]. Using MDA, the three-phase learning cutoff points were after 33 and 72 cases, and after 44 and 78 cases in two robotic rectal surgery studies; and after 44 and 90 cases for robotic right hemicolectomy surgery. These analyses can analyse the learning curve to reflect and adjust for surgical outcomes. There was variability in how MDA was performed in studies. Some studies calculated risk adjusted CUSUM graphs using univariate analysis of risk factors and multifactorial logistic regression to calculate probability of failure, with failure and success assigned scores of 1 and 0, respectively. Odermatt et al. analysed CUSUM graphs for lymph node harvest, length of stay and major complications, using the surgeon’s matched laparoscopic reference group as a baseline [17].

The learning curve has been demonstrated to be longer for recovery and safety metrics (LOS, complications). Improvements in these variables may continue for extended periods after the learning curve for operation time has been overcome [2]. Several studies defined surgical failure by occurrence of any of the selected parameters: open conversion, positive resection margin, harvested lymph nodes less than 12, morbidity or mortality and local recurrence [11, 34]. In assessing learning curves, the “failure”, conversion and complication rates should be reported overall and ideally improvements over the three time-based phases should be demonstrated. In RCS studies, the conversion rate ranged from 0 to 11% [8, 10, 11, 14, 15, 17–19, 21, 31]. The anastomotic leak rate ranged from 0 to 11% [8, 10, 11, 13, 14, 18, 19, 21]. Four studies reported more (1) and equivalent (3) Clavien–DIndo [35] grade III or IV complications in phase 3 [11, 14, 18, 19]. This may be related to selection of more complicated cases or indicate a need for more cases to achieve proficiency. In addition, there may be a relationship between open conversions and worse patient outcomes. Surgeon inexperience has been reported to be a predictive risk factor for open conversion [36].

Bokkari et al. proposed three important factors for surgeons to achieve to attain RCS mastery: overcoming lack of tactile feedback with compensation by visual cues, keeping track of robotic instrument positions when they are not in the field of vision and optimising maneuverability of the robotic arms while operating at the console without direct vision [7]. Overall assessment of surgeon robotic skills may be a more reliable method to assess learning. Global Evaluative Assessment of Robotic Skills (GEARS), modelled after global rating scales for open and laparoscopic surgery, has been validated as a clinical skills assessment tool. GEARS consists of six domains (depth perception, bimanual dexterity, efficiency, force sensitivity, autonomy and robotic control) scored on a 5-point Likert scale and has been used to classify surgeon expertise levels [37, 38]. The assessment of these skill domains may not be as applicable for robotic surgery compared with laparoscopic surgery because of technological factors. Depth perception (3-dimensional visualisation) provided by the robotic console has been shown to reduce task performance time and error rates [39]. Robotic assistance has also been shown to improve the fine motor skills of the nondominant hand and confer virtual ambidexterity [40]. Conversely, lack of haptic feedback can result in excessive or inadequate application of force, causing damage or slippage of tissues [41].

## Learning curve associated with other robotic surgery

The learning curves to gain proficiency based on time improvements with robotic urological, gynaecological and upper gastrointestinal surgery were similar to RCS studies. Studies have indicated that surgeons can perform robot-assisted prostatectomy, cystectomy and upper tract urological surgery safely after 30–40 cases [42, 43]. Flattening of the learning curve of robotic hysterectomy based on operative time occurred after 20–30 cases [44, 45]. More complicated surgery may be associated with a longer time-based learning curve. 150 robot-assisted partial nephrectomy cases were required before no further improvement in warm ischaemia time was observed [46]. The learning curve was longer if assessment of functional or oncological outcomes were assessed [42, 47]. In one study, robotic radical prostatectomy surgery scores surpassed open surgery scores for sexual function after 99 cases, for urinary incontinence after 182 cases and for positive T2 surgical margin after 108 cases. [47].

One study indicated that previous experience as a bedside assistant for robotic prostate surgery was associated with a significantly shorter surgery console time when the assistant progressed to be the primary surgeon [48]. A period of robotic hysterectomy assistance (15 cases) before commencing robot-assisted hysterectomy as the surgeon was also shown to bypass the time-based learning curve [44]. The benefit may relate to experience gained on collisions and trouble-shooting management from observations during their time as bedside assistant. One study investigating the impact of simulator training included assessment of supra-cervical hysterectomies on real patients: 14 novice robotic surgeons who trained on a virtual simulator for an average of 20 h outperformed (with regard to time, blood loss and blinded video assessment) a control group with no simulator exposure [49]. The learning curve may be shortened by pre-operative warm-up on a simulator, as well as sequentially learning different steps of an operation until proficient before moving on to more complex steps [42]. Mentorship and formal robotic proficiency skills curriculum for robotic pancreaticoduodenectomy (divided into seven steps of resection and reconstruction) was found to be associated with reduced operating time and complication rate [50].

## Conclusion

Procedural skills attained during open and laparoscopic colorectal surgery are transferable to robotic surgery. Experienced colorectal surgeons can perform robotic surgery safely, even on patients with high complexity early in the learning curve. Case complexity rather than case number may have the most impact on operation time. Analysis of patient outcome and/or GEARS may be more reliable for learning curve assessment.
